# Constructing an evaluation index system for clinical nursing practice teaching quality using a Delphi method and analytic hierarchy process-based approach

**DOI:** 10.1186/s12909-024-05770-y

**Published:** 2024-07-19

**Authors:** Shengxiao NIE, Lei WANG

**Affiliations:** Department of Nursing, Beijing Hospital, National Center of Gerontology; Institute of Geriatric Medicine, Chinese Academy of Medical Sciences, No.1 Dahua Road, Dongcheng District, Beijing, 100730 People’s Republic of China

**Keywords:** Clinical nursing, Teaching quality, Evaluation, Index system, Delphi survey, Analytic Hierarchy Process (AHP)

## Abstract

**Background:**

The key step in evaluating the quality of clinical nursing practice education lies in establishing a scientific, objective, and feasible index system. Current assessments of clinical teaching typically measure hospital learning environments, classroom teaching, teaching competency, or the internship quality of nursing students. As a result, clinical evaluations are often insufficient to provide focused feedback, guide faculty development, or identify specific areas for clinical teachers to implement change and improvement. Therefore, the purpose of our study was to to construct a scientific, systematic, and clinically applicable evaluation index system of clinical nursing practice teaching quality and determine each indicator’s weight to provide references for the scientific and objective evaluation of clinical nursing practice teaching quality.

**Methods:**

Based on the “Structure-Process-Outcome” theoretical model, a literature review and Delphi surveys were conducted to establish the evaluation index system of clinical nursing practice teaching quality. Analytic Hierarchy Process (AHP) was employed to determine the weight of each indicator.

**Results:**

The effective response rate for the two rounds of expert surveys was 100%. The expert authority coefficients were 0.961 and 0.975, respectively. The coefficient of variation for the indicators at each level ranged from 0 to 0.25 and 0 to 0.21, and the Kendall harmony coefficients were 0.209 and 0.135, respectively, with statistically significant differences (P < 0.001). The final established index system included 3 first-level, 10 second-level, and 29 third-level indicators. According to the weights computed by the AHP, first-level indicators were ranked as “Process quality” (39.81%), “Structure quality” (36.67%), and “Outcome quality” (23.52%). Among the secondary indicators, experts paid the most attention to “Teaching staff” (23.68%), “Implementation of teaching rules and regulations (14.14%), and “Teaching plans” (13.20%). The top three third-level indicators were “Level of teaching staff” (12.62%), “Structure of teaching staff” (11.06%), and “Implementation of the management system for teaching objects” (7.54%).

**Conclusion:**

The constructed evaluation index system of clinical nursing practice teaching quality is scientific and reliable, with reasonable weight. The managers’ focus has shifted from outcome-oriented to process-oriented approaches, and more focus on teaching team construction, teaching regulations implementation, and teaching design is needed to improve clinical teaching quality.

## Background

As an extension of school teaching, clinical nursing practice teaching is an essential constituent of nursing education, as it is a vital link to cultivating students’ practical ability, and it plays a pivotal role in employment choice, career development, and professional quality of nursing students [1]. Evaluation is a form of action research committed to creating changes in the evaluated process by offering applicable recommendations [2]. Based on evaluation results, the original teaching plans and activities can be adjusted timely. This ensures effective quality at every stage of the teaching process, guaranteeing overall teaching quality and continuous improvement.

The critical step in evaluating the quality of clinical nursing practice education lies in establishing a scientific, objective, and feasible index system based on evaluation objectives. Evaluation of teaching without having effective teaching indicators not only does not improve the quality of instruction but also causes quality fall [3]. Evaluations in the clinical teaching are fraught with problems. Current assessments of clinical teaching typically measure hospital learning environments [4, 5], classroom teaching [6], or the internship quality of nursing students [7, 8]. As a result, clinical evaluations are often insufficient to provide focused feedback, guide faculty development, or identify specific areas for clinical teachers to implement change and improvement [9].

In recent years, major national reforms of postgraduate medical education have taken place in numerous countries, including reforms about the requirements of teaching and assessment strategies [10]. China is no exception. As an interdisciplinary subject, medical education is related to the implementation of the “Healthy China” strategy [11]. With the rapid development of the nursing profession, hospitals in China undertake teaching tasks beyond internships. These tasks include instructing nursing interns, nurses attending advanced studies and specialist nurse trainees, continuing education for nurses, and other training and assessment work. Therefore, the existing index system cannot comprehensively evaluate the quality of clinical nursing practice teaching.

Therefore, the purpose of our study was to construct a scientific, systematic, and clinically applicable evaluation index system of clinical nursing practice teaching quality and determine the weight of each indicator based on the “Structure-Process-Outcome” model to provide references for the scientific and objective evaluation of clinical nursing practice teaching quality.

## Methods

### Construction of the evaluation index system of clinical nursing practice teaching quality

#### Establish a study group

A research group is composed of 7 nursing managers and nursing experts. Among these, one has a senior professional title (Deputy Director of Nursing Department, in charge of clinical nursing practice education), one has an associate senior professional title, two have an intermediate professional title, and three are nursing undergraduate students.

The main tasks of this research group were as follows: responsible for reviewing the literature, producing a first draft of the key indicators, establishing an expert inquiry form, determining the consulting experts, collecting their views and opinions on the indicator system, and statistical analysis.

#### Conceptual model

To evaluate the quality of clinical nursing practice teaching, we used the “Structure—Process—Outcome” framework described by Donabedian [12]. His three-part approach makes quality assessment possible, assuming structure (e.g., attributes of material or human resources and organizational structure) influences process (what is done in giving and receiving care), which influences outcome (e.g. health status) [12]. We chose Donabedian’s model as it is widely used and allows both researchers and policymakers to conceptualize the underlying mechanisms that may contribute to poor quality of clinical practice nursing teaching.

#### Construct consultation questionnaire

A systematic literature search was performed using PubMed, Medline, CNKI, VIP, and Wangfang databases from the inception of each database to December 2020. The following main search terms were used: “analytic hierarchy process (AHP)”, “Delphi method”, “clinical nursing”, “quality of teaching”, “clinical education”, “indicator” and “indicator system”. Based on Donabedian’s model, the study group generated an original draft of the evaluation indicator system consisting of 3 first-level, 10 second-level, and 28 third-level indicators. The initial draft was verified for readability and feasibility by two education experts.

Delphi expert consultation questionnaire included three parts: an explanation of the questionnaire, basic information of experts, and the main text of the questionnaire: (1) Explanation of the questionnaire, included the research background, purpose, and meaning; (2) Basic information of experts, included the expert’s age, education background, position, professional title, years of experience in nursing teaching and management, the degree of the expert’s familiarity with the indicators (Cs) and the educational level of the expert and the basis for judgment (Ca); (3) Main text of questionnaire, included the content of each evaluation indicator, and the method of scoring the importance of each item. The experts were required to rate each item on a five-point Likert scale from 1 (unimportant) to 5 (very important), give comments, and suggest additional items.

#### Selection of the experts

The number of consultation experts was usually between 15 and 50 [13]. Moreover the more experts there were, the more reliable the result would be. In our study, the purposeful sampling method was used to select 18 experts from 4 tertiary hospitals and 2 nursing schools in Beijing as consulting experts, including 14 clinical nursing practice teaching experts (77.8%) and 4 academic nursing teaching management experts (22.2%). The inclusion criterion of the experts in this study was: (a) bachelor’s degree or above, intermediate professional title or above; (b) engaged in clinical nursing practice teaching, clinical nursing teaching management in tertiary hospitals, or nursing education for at least ten years; (c) informed consent, actively participates in this study and able to guarantee to complete two rounds of questionnaires.

The panel of experts in this study were aged between 35 and 62 years old (mean 44.44 ± 6.93), engaged in clinical nursing practice teaching for 12- 42 years (mean 22.56 ± 7.91), or in nursing teaching management for 9–36 years (mean 17.33 ± 7.84). There were 4 experts with senior professional titles (22.2%), 11 with associate senior professional titles (61.1%), and 3 with intermediate professional titles (16.7%). Among these, 3 had doctoral degrees (16.7%), 7 had master’s degrees (38.9%), and 8 had bachelor’s degrees (44.4%). All of them hold leadership positions in nursing teaching in the department.

#### Conduct expert consultation

In Jan 2021, the research group launched the first round of Delphi consultation with the selected experts. The consultation questionnaire was sent via WeChat or email to the experts. After collecting the first round of questionnaires, the index items were analyzed concerning expert opinions. The index items were analyzed according to the criteria that the coefficient of variation should be less than 0.25, the mean should be greater than 3.5, and the full score rate should be above 20% [8]. If the coefficient of variation is significant, it indicates a disagreement among experts on the item. Based on the first round of consultation, items were deleted, modified, and added to form the second round of consultation questionnaire. In March 2021, the second round of consultation questionnaires was carried out. The experts were also given two weeks to fill in the questionnaires. In the second round of Delphi consultation, the experts reached a consensus, and all the index items met the selection criteria.

### Construct and conduct expert judgment matrix

To understand the relative importance of each indicator, we adopted the Analytic Hierarchy Process (AHP) developed by Saaty to construct an expert judgment matrix [14, 15]. According to Triantaphyllou and Mann, “the AHP is a decision support tool which uses a multi-level hierarchical structure of objectives, criteria, subcriteria, and alternatives. The pertinent data are derived by using a set of pairwise comparisons. These comparisons are used to obtain the weights of importance of the decision criteria, and the relative performance measures of the alternatives in terms of each individual decision criterion” [16].

The questionnaires were presented by paired indicators. For each question, both sides had a factor, and the more important one was selected first by the professionals. Then, the score scale of the relative importance (1–9) was determined. In this scale, 1, 3, 5, 7, and 9 corresponded to “equally important,” “slightly more important,” “moderately more important,” “strongly more important,” and “absolutely more important,” respectively, while 2, 4, 6, and 8 represented importance levels between adjacent levels. A sample question is (What do you think about the relative importance of the two structure indicators [Conditions of the department and teaching staff]? Please check the proper field and then determine the relative importance.)

In May 2021, we invited the experts who participated in the second round to complete a comparison matrix. The experts were also given two weeks to fill in the questionnaires.

### Data analysis

Data were analyzed using SPSS version 16.0, and the degree of experts’ activity was expressed by the questionnaire response rate. The self-evaluation standard of expert familiarity (Cs) was to assign values to each entry and finally calculate the arithmetic mean based on the assignment method of Cs = 0.9 (very familiar), Cs = 0.7 (familiar), Cs = 0.5 (generally familiar), Cs = 0.3 (less familiar), Cs = 0.1 (very unfamiliar). Experts’ judgment basis (Ca) was that they divided the degree of influence of factors that affected problem judgment into large-, medium-, and small-level. Then they assigned values to the different influence degrees as follows: theoretical analysis (0.3, 0.2, 0.1), practical experience (0.5, 0.4, 0.3), understanding of domestic and foreign counterparts (0.1, 0.1, 0.1), intuition (0.1, 0.1, 0.1). Finally, the arithmetic mean was calculated. The authority coefficient of the experts (Cr) was determined by the coefficient of judgment basis (Ca) and the coefficient of familiarity (Cs), calculated as Cr = (Ca + Cs)/2, and the results are acceptable when Cr > 0.7 [17]. The degree of expert opinion concentration was expressed by the full score rate, mean, and standard deviation of item importance. The coefficient of variation (CV) was used to assess the consistency of the experts’ opinions concerning the indicators. Besides, we used Kendall’s coefficient of concordance (Kendall’s W) to test the consistency of experts’ opinions.

AHP was used to calculate the weight of each indicator, and the consistency ratio < 0.1 was considered a reasonable weight distribution [7].

## Results

### The enthusiasm of the experts

In the first and second rounds of expert consultation, 18 questionnaires were sent out, and 18 questionnaires were effectively recovered. The effective recovery rate of the expert consultation form was 100.0%. In the first round, 12 experts put forward some suggestions on the evaluation index of clinical nursing practice teaching, accounting for 66.7%. In the second round, 8 experts put forward relevant suggestions on the evaluation index of clinical nursing practice teaching, accounting for 44.4%. It is generally believed that the effective recovery rate of the questionnaire is more than 70%, indicating that the experts in this study have high enthusiasm and great interest in this field [18].

### Expert authority

The judgment coefficient (Ca) was 0.944, the familiarity coefficient (Cs) was 0.978, and the authority coefficient (Cr) was 0.961 in the first round. The judgment coefficient (Ca) was 0.950, the familiarity coefficient (Cs) was 1.000, and the authority coefficient was (Cr) 0.975 in the second round, indicating a high degree of authority.

### Concentration degree of expert opinions

The concentration degree of expert opinions in this study was mainly represented by the average score of importance (X), the standard deviation of the score of importance (S), and the ratio of full-score K (%). In the first round, the X of the 41 indicators was 4.22 to 5.00, the S was 0.00 to 1.07, and the K was 50.0% to 100.0%. In the second round, the X of the 42 indicators was 4.39 to 5.00, S was 0.00 to 0.92, and K was 66.1% to 100%. These data showed that the concentration degree of expert opinions was highly concentrated.

### Coordination degree of expert opinions

The coordination degree of expert opinions was measured using the coefficient of variation and the Kendall Coordination Coefficient (W). The coefficient of variation of indicators in round 1 was 0 to 0.25. The coefficient of variation of indicators in round 2 was 0 to 0.21. The Kendall coordination coefficients of the 2 Delphi surveys were 0.209 and 0.135, respectively (*P* < 0.05), indicating that the degree of expert coordination was good (Table 1).

**Table 1:** Coordination degree of expert opinions

Hierarchical level	Round 1				Round 2			
	N	Kendall’ W	χ2	P	N	Kendall’ W	χ2	P
First-level indicators	3	0.211	7.600	0.022	3	0.142	94.881	< 0.001
Second-level indicators	10	0.242	39.251	< 0.001	10	0.149	24.195	0.004
Third-level indicators	28	0.198	100.015	< 0.001	29	0.136	68.352	< 0.001
All	41	0.209	154.008	< 0.001	42	0.135	99.646	< 0.001

Kendall’W, Kendall’s coefficient of concordance

### The result of expert consultation

In round 1, 12 experts suggested amendments to some indicators. During the discussion, some indicators were amended by the study group as follows. There are three first-level and ten second-level indicators, with the number unchanged. The “regulations” in the second-level indicators was changed to “implementation of teaching rules and regulations”. Third-level indicators were screened as follows: (1) Delete “teaching arrangement” “specially-assigned person in charge of teaching” and “the role of head nurses in teaching management”; (2) Add “targeted teaching plans” “evaluation of teaching process” “nursing competence of nursing interns” and “achievements of teaching related scientific research”; (3) Two indications “implementation of continuing nursing education” and “teaching situation within the department” were merged into one indicator “implementation of teaching plans within the department”.

**Fig. 1:**
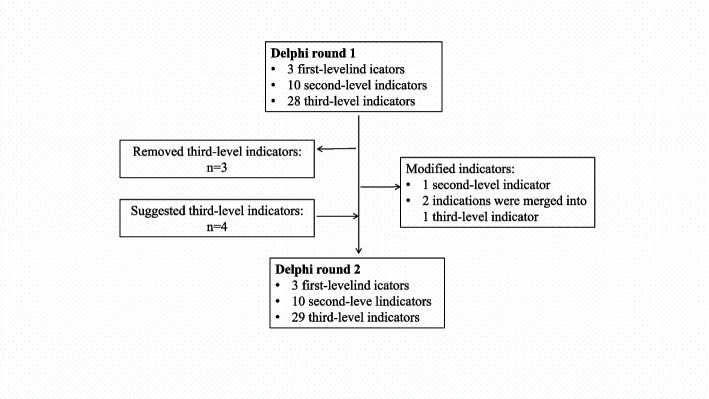
Flow diagram of the Delphi process

In round 2, the consulting experts only revised some of the wording. The expert panel then achieved consensus about the final indicator system, which consists of three 3 first-level, 10 second-level, and 29 third-level indicators, as shown in Table 2. A study flow diagram of the Delphi process is shown in Fig. 1.

**Table 2:** Evaluation index system of clinical nursing practice teaching quality

Indicators (Code)	Descriptions	x ± s	CV	K(%)
**(A1) Structure quality**		5.00 ± 0.00	0.000	100
(B1) Conditions of the department		4.78 ± 0.55	0.115	83.3
(C1) Basic conditions of the department	Number of beds; Types of diseases and number of patients in the department; Radio of nurse/ bed; Discipline status of the department (such as being approved as a national critical clinical specialty)	4.72 ± 0.58	0.122	77.8
(C2) Teaching conditions of the department	Separate teaching place (such as demonstration classroom); Teaching equipment (such as multimedia, projector, etc.); Teaching aids (such as simulator); Teaching materials	4.83 ± 0.51	0.106	88.9
(C3) Teaching atmosphere of the department	Harmonious relationship between medical staff in the department; Strong teaching awareness among nursing staff in the department	5.00 ± 0.00	0.000	100
(B2) teaching staff		4.94 ± 0.24	0.048	94.4
(C4) Structure of teaching staff	Education background of teaching staff; Title of teaching staff; Years of working experience of teaching staff; Years of teaching experience of teaching staff; Ratio of teacher staff and students of different types; Experiences of training of teaching staff; Teaching qualifications of teacher staff (such as in universities, etc.)	4.83 ± 0.38	0.079	83.3
(C5) Level of teaching staff	Professional ethics (professional values, personal qualities, etc.); Professional knowledge; Clinical competence; Teaching ability; Humanistic care ability; Scientific research ability	5.00 ± 0.00	0.000	100
**(A2) Process quality**		5.00 ± 0.00	0.000	100
(B3) Implementation of teaching rules and regulations		4.89 ± 0.32	0.066	88.9
(C6) Implementation of the management system for teachers	Implementation of the selection rules for teachers; Implementation of job management regulations for teachers; Implementation of the incentive rules for teachers; Implementation of evaluation system for teachers; Implementation of training system for teachers	4.89 ± 0.23	0.066	88.9
(C7) Implementation of the management system for teaching objects	Implementation of management systems for different teaching objects; Implementation of continuing education management system for nurses; Implementation of the multi-level training system for nurses	4.78 ± 0.43	0.090	77.8
(B4) Teaching plans		5.00 ± 0.00	0.000	100
(C8) Targeted teaching plans	Teaching plans for different teaching objects; Teaching plans for multi-level nurses in the department; Teaching plans for nursing talent development	4.94 ± 0.24	0.048	94.4
(C9) Teaching plans can meet the training requirements	Teaching plans can meet the training requirements of the school/social organization/hospital; Teaching plans can meet the needs of professional development; Teaching plans can meet the learning needs of the teaching object	4.94 ± 0.24	0.048	94.4
(C10) Appropriate teaching plans	From simple to deep teaching content; Practical teaching content; Advanced teaching content; Feasible teaching plans	4.94 ± 0.24	0.048	94.4
(B5) Implementation process of teaching plan		4.94 ± 0.24	0.048	94.4
(C11) Teaching arrangements	Specially assigned person in charge of teaching; Reasonable shifts for teaching objects; Providing opportunities for teaching objects to participate in nursing rounds, doctor rounds, department lectures, etc.; Providing practical opportunities for teaching objects; Educating someone according to his natural ability; Reasonable application of multiple teaching methods; Evaluating teaching objects on time; Summarizing the teaching situation on time; Able to achieve teaching objectives according to the teaching plan; Directing teaching objects to write papers	4.83 ± 0.51	0.106	94.4
(C12) Implementation of teaching plans within the department	Implementation rate of national/provincial/district level continuing education programs; Reporting the implementation situations of national/provincial/district level continuing education programs to higher authorities on time; Annual compliance rate of continuing education for nurses in the department; Learning situation within the department (including frequency and participation); Nursing rounds within the department (including frequency and participation); Assessment of knowledge and skills within the department (including frequency and participation)	4.78 ± 0.65	0.135	88.9
(C13) Implementation of teaching plans for nursing talent development within the department	Specialist nurses trained this year; Nurses attending further education in higher-level hospitals; Nurses attending further education in other departments within the hospital	4.88 ± 0.50	0.103	88.9
(C14) Evaluation of teaching process	Supervision of the teaching process by the head nurse or teaching management personnel; Evaluation of teaching objects by teacher on time; Evaluation of teacher or department by teaching objects on time; Continuous improvement of teaching quality	4.94 ± 0.24	0.048	94.4
(C15) Records of teaching process	Records of teaching objects’ information; Records of talent personnel development; Records of teaching activity (lectures, rounds, etc.); Records of teaching and research achievements	4.72 ± 0.46	0.098	72.2
**(A3) Outcome quality**		4.94 ± 0.24	0.048	94.4
(B6) Annual teaching workload		4.78 ± 0.55	0.115	83.3
(C16) Number and duration of different teaching objects accepted by the department	Number of different teaching objects accepted by the department (nursing interns, advanced training nurse, specialist nurse trainees, new nurses, rotating nurses, standardized training, etc.); Learning duration of different teaching objects accepted by the department (nursing interns, advanced training nurse, specialist nurse trainees, new nurses, rotating nurses, standardized training, etc.)	4.81 ± 0.54	0.113	83.3
(C17)Teaching workload	Implementation of national/provincial continuing education training courses; Numbers and duration of lectures for national/provincial continuing education programs; Number of lectures for district-level continuing education programs; Numbers and duration of other lectures undertaken within the hospital (pre-job training, temporary lectures, etc.); Numbers and duration of teaching at universities	4.78 ± 0.43	0.090	77.8
(C18) Workload of examination	Examination work from the nursing department; Examination work for specialist nurses trainees from the Chinese Nursing Association, Provincial Nursing Association, and other academic institutions	4.39 ± 0.92	0.209	61.1
(B7) Examination scores of teaching subjects		4.94 ± 0.24	0.048	94.4
(C19) Knowledge scores of teaching objects	Scores of basic knowledge; Scores of specialized knowledge	4.83 ± 0.38	0.079	83.3
(C20) Skill scores of teaching objects	Scores of basic skills; Scores of specialized skills	4.89 ± 0.32	0.066	88.9
(B8) Nursing competence of the teaching object		4.94 ± 0.24	0.048	94.4
(C21) Nursing competence of new nurses and standardized training nurses	Consultation skills; Physical examination skills; Humanistic care/professional competence; Clinical judgment ability; Professional consulting, advice, and communication skills; Organizational ability and efficiency	4.94 ± 0.24	0.048	94.4
(C22) Nursing competence of nursing interns	Consultation skills; Physical examination skills; Humanistic care/professional competence; Clinical judgment ability; Professional consulting, advice, and communication skills; Organizational ability and efficiency	4.83 ± 0.38	0.079	83.3
(C23) Nursing competence of nurses attending advanced studies	Clinical application capabilities of new technologies and services; Educational capabilities of new technologies and services	4.89 ± 0.32	0.066	88.9
(C24) Nursing competence of specialist nurses trainees	Clinical knowledge and practical ability; Teaching ability; Consulting ability; Scientific research ability	4.89 ± 0.32	0.066	88.9
(B9)Teaching evaluation		4.94 ± 0.24	0.048	94.4
(C25) Satisfaction of teaching objects with the department/teaching staff	Satisfaction of the teaching object with the teacher; Satisfaction of the teaching object with the teaching arrangements; Satisfaction of the teaching object with the learning gains	4.83 ± 0.38	0.079	83.3
(C26) Evaluation of teaching managers on teachers	Completion of teaching tasks; Head nurse’s evaluation of the teaching quality of teachers; Adverse nursing events, nursing errors, and accidents of teaching objects	4.89 ± 0.32	0.066	88.9
(B10) Teaching achievements		4.61 ± 0.61	0.132	66.7
(C27) Achievements of teaching related scientific research	Approved scientific research projects; Papers; Patents; Published Books; Award of teaching related scientific research achievement	4.56 ± 0.78	0.170	66.7
(C28) Academic training or conferences of teachers attended	Oral presentation, lecture, or host at academic conferences; Posters at academic conferences; Awards at academic conferences; Teaching-related training of teachers attended	4.61 ± 0.70	0.151	72.2
(C29) Awards related to teaching	Advanced awards of teachers; Awarded as excellent students of teaching objects; Awards of teachers and students participating in various nursing competitions and activities	4.67 ± 0.69	0.147	77.8

*CV* Coefficient of variation, *K* The ratio of full-score

### Analysis of matrix and weight of indicators

#### Analysis of matrix and weight of the first-level indicators

Matrix and weight analysis of the first-level indicators (Structure quality, Process quality, and Outcome quality) are shown in Table 3. Among the three first-level indicators of the evaluation index system of clinical nursing practice teaching quality, “Process quality”(A2) was perceived as more important than “Structure quality”(A1) and “Outcome quality”(A3). The relative contribution was 39.81%, 36.67%, and 23.52%, respectively.

**Table 3:** Analysis of matrix and weight of the first-level indicators

First-level indicators	A1	A2	A3	Weight	Rank
(A1) Structure quality	1	0.949	1.514	36.67%	2
(A2) Process quality	1.054	1	1.744	39.81%	1
(A3) Outcome quality	0.661	0.574	1	23.52%	3

CI < 0.001

#### Analysis of matrix and weight of second-level indicators

The relative weights of second-level indicators are shown in Table 4. The second-level indicators analysis showed that in the “Structure quality”, “Teaching staff” (B2) was perceived as more important than “Conditions of the department” (B1) (within-dimensional weight: 64.57% and 35.43%, respectively). Among the sub-dimensions of “Process quality”, the relative importance was “Implementation of teaching rules and regulations” (B3) (35.53%), followed by “Teaching plans” (B4) (33.15%), and “Implementation process of teaching plans”(B5) (31.32%). Among the sub-dimensions of “Outcome quality”, the relative importance was “Nursing competence of the teaching object” (B8) (28.40%), followed by “Examination scores of teaching subjects” (B7) (21.19%), “Annual teaching workload” (B6) (20.05%), “Teaching evaluation” (B9) (16.17%), and then “Teaching achievements” (B10) (14.19%).

**Table 4:** Analysis of matrix and weight of the second-level indicators

First-level indicators	Second-level indicators						Weight	Overall weight	Rank	CI
Structure quality		B1	B2							0
	(B1) Conditions of the department	1	0.549				35.43%	12.99%	2	
	(B2) Teaching staff	1.822	1				64.57%	23.68%	1	
Process quality		B3	B4	B5						0
	(B3) Implementation of teaching rules and regulations	1	1.051	1.156			35.53%	14.14%	1	
	(B4) Teaching plans	0.951	1	1.038			33.15%	13.20%	2	
	(B5) Implementation process of teaching plans	0.865	0.963	1			31.32%	12.47%	3	
Outcome quality		B6	B7	B8	B9	B10				0.010
	(B6) Annual teaching workload	1	1.103	0.698	1.167	1.328	20.05%	4.72%	3	
	(B7) Examination scores of teaching subjects	0.907	1	0.997	1.295	1.297	21.19%	4.98%	2	
	(B8) Nursing competence of the teaching object	1.432	1.003	1	2.175	2.108	28.40%	6.68%	1	
	(B9) Teaching evaluation	0.857	0.772	0.46	1	1.313	16.17%	3.80%	4	
	(B10) Teaching achievements	0.753	0.771	0.474	0.762	1	14.19%	3.34%	5	

#### Weight analysis of third-level indicators

The relative weights of third-level indicators are shown in Table 5. The 10 third-level indicators with the highest ranks were: 1) level of teaching staff (C5); 2) structure of teaching staff (C4); 3) implementation of the management system for teaching objects (C7); 4) Implementation of the management system for teachers (C6); 5) targeted teaching plans (C8); 6) teaching conditions of the department (C2); 7) teaching plans can meet the training requirements (C9); 8) teaching atmosphere of the department (C3); 9) basic conditions of the department (C1); and 10) teaching arrangements (C11). The top 1, 2, 6, 8, and 9 indicators fell into the “Structure quality” dimension; numbers 3–5, 7, and 10 into the “Process quality” dimension.

**Table 5:** Weight analysis of third-level indicators

Codes of First-level indicators	Codes of Second-level indicators	Third-level indicators				
		Codes	Weight	Rank	Overall weight	Overall rank
A1	B1	C1	28.60%	3	3.72%	9
		C2	37.22%	2	4.84%	6
		C3	34.18%	1	4.44%	8
	B2	C4	46.72%	2	11.06%	2
		C5	53.28%	1	12.62%	1
A2	B3	C6	46.72%	2	6.61%	4
		C7	53.28%	1	7.54%	3
	B4	C8	42.26%	1	5.58%	5
		C9	34.71%	2	4.58%	7
		C10	23.03%	3	3.04%	
	B5	C11	25.81%	1	3.22%	10
		C12	24.16%	2	3.01%	
		C13	18.79%	4	2.34%	
		C14	21.59%	3	2.69%	
		C15	9.65%	5	1.20%	
A3	B6	C16	49.31%	1	2.33%	
		C17	32.34%	2	1.53%	
		C18	18.35%	3	0.87%	
	B7	C19	48.68%	2	2.43%	
		C20	51.32%	1	2.56%	
	B8	C21	33.86%	1	2.26%	
		C22	17.62%	4	1.18%	
		C23	18.59%	3	1.24%	
		C24	29.93%	2	2.00%	
	B9	C25	63.21%	1	2.40%	
		C26	36.79%	2	1.40%	
	B10	C27	43.03%	1	1.44%	
		C28	29.84%	2	1.00%	
		C29	27.13%	3	0.91%	

## Discussion

The evaluation index system of clinical nursing practice teaching quality constructed in this study is scientific. First, it is based on the Structure-Process-Outcome quality structure model as a theoretical model, and the practice shows that the theory is very mature in establishing evaluation indicators of clinical nursing teaching quality [7]. Second, the quality evaluation indicator system is established based on an extensive literature review, Then, the content of the index system is finally formed through two rounds of the Delphi method. Finally, the Analytic Hierarchy Process was utilized to determine the weights of each hierarchical indicator, and the consistency ratios for all levels were below 0.1, indicating a reasonable allocation of weights for the indicators.

The reliability of the research results is closely related to the representativeness, enthusiasm, authority, and consistency of the consulted experts [19]. The representativeness of the selected experts determines the authority of the research results [17]. In this study, the 18 selected experts were experienced clinical nursing teaching management experts from hospitals or higher education institutions.Besides, these experts have been engaged in clinical nursing practice teaching or nursing teaching management for more than 15 years, and all hold leadership positions in nursing teaching in the department. Furthermore, 15 experts (83.3%) had associate senior or higher professional titles. Therefore, the expert panel in our study was well structured and possessed rich theoretical knowledge and practical experience in the research field, making their opinions highly representative. The effective response rate for both rounds of consultation was 100%, and the percentages of experts providing modification suggestions were 66.7% and 44.4%, respectively, indicating a high level of enthusiasm from the experts. The expert authority coefficients for both rounds were greater than 0.9, and considering their professional titles and educational backgrounds, the experts demonstrated a high level of authority. The Kendall’s coefficient of concordance for expert opinions was 0.209 and 0.135 for the two rounds, respectively, with a *p*-value < 0.001, indicating that the weight distribution of indicators at all levels is reasonable.

The unique contributions of our research is that we have incorporated as many indicators as possible related to clinical nursing practice teaching quality and established a comprehensive evaluation system from the perspective of clinical nursing teaching managers. This can be reflected in the following three aspects. First, we include not only indicators related to outcome quality but also indicators related to structure and process quality. Second, the index system we established includes both quality and quantity indicators. Finally, the index system we established is not used to evaluate specific teaching objects but to evaluate all teaching objects comprehensively. These are quite different from previous studies, which focused more on the competency assessment for clinical nursing teachers [20, 21] and the clinical skills of students [22, 23]. However, clinical nursing teachers’ and students’ competency cannot fully reflect the quality of clinical nursing practice teaching. As pointed out by Xu et al. [24], nursing education managers’ standardized and systematic supervision, management, and evaluation of clinical teaching quality is crucial to ensure the quality of clinical nursing teaching. The evaluation index system in our study was established from the perspective of clinical nursing teaching managers and can meet the rapid development of teaching task assessment requirements.

Similar to other findings [1, 25, 26], the “Process quality” (39.81%) has the highest weight among the first-level indicators. This reflects a shift in the managers’ focus from outcome-oriented to process-oriented approaches. Among all the third-level indicators, “Implementation of the management system for teaching objects” and “Implementation of the management system for teachers” ranked 3 and 4, and fell into the second-level indicator “Implementation of teaching rules and regulations”, indicating the importance of teaching rules and regulations. Teaching rules and regulations are the foundation for the orderly operation of clinical nursing teaching. In recent years, management rules and regulations for teachers have been gradually developed or improved, including selection systems, work systems, incentive systems, evaluation and assessment systems, training systems, and management systems for teaching objects [27–30]. Implementing these regulations directly affects whether the qualifications of teachers meet the regulations, the standardization and enthusiasm of teaching work, the quality of teaching objects, and the achievement of educational goals. Therefore, the weight value for these indicators is relatively high. We also found that “Targeted teaching plans” and “Teaching plans can meet the training requirements” ranked 5 and 7, and “Teaching arrangements” ranked 10 and fell into the second-level indicator “Implementation process of teaching plans”, which reflects the importance that teaching managers attach to the teaching plan. It is suggested that teaching plans should be tailored to different teaching objects, objectives, and learning durations, and appropriately arranged.

We found that “Level of teaching staff” and “Structure of teaching staff” ranked 1 and 2 and fell into the second-level indicator “Teaching staff”. This indicates that teaching faculty is crucial in the clinical nursing practice teaching quality evaluation index system. While care for patients must be the top priority for healthcare workers, universities must also ensure that teaching can be adequately delivered [30]. Therefore, faculty development is key to ensuring quality clinical teaching [31]. Clinical teachers face different challenges as they are expected to produce high-impact research, contribute to medical education, and deliver high-quality patient care, virtually all simultaneously [32]. Currently, undergraduate nursing and master’s education in China are developing rapidly, and teaching requirements and standards are becoming increasingly high. Moreover, the variety and quantity of specialized nurse training are constantly increasing, which also puts forward high requirements for the teaching level of clinical nursing teachers [33]. As teaching managers, on the one hand, we should select high-level teachers. On the other hand, we should strengthen training to improve the nursing team’ overall quality and clinical nursing teaching outcomes.

Our findings demonstrated that “Conditions of the department” is a crucial indicator of clinical nursing practice teaching quality. As shown in Table 5, “Basic conditions of the department”, “Teaching conditions of the department” and “Teaching atmosphere of the department” ranked high among all indicators, at 9th, 6th, and 8th respectively. These three indicators belong to the “Conditions of the department” indicator. Although the importance of teaching conditions for teaching quality has been fully recognized [34, 35], poor teaching conditions appear to be an international problem identified quite some time ago [30, 35]. Basic conditions of the department, including number of beds, types of diseases and number of patients in the department, radio of nurse/ bed, and discipline status of the department, reflect the level of discipline and the level of busyness of nursing work. A department with high-level development disciplines exposed teaching subjects to more advanced technologies. Clinical teachers in a busy department may not have enough time to engage in teaching activities, while lack of time is a significant barrier to planning and delivering good clinical teaching [30]. Teaching conditions of the department, including separate teaching places, teaching equipment, teaching aids (such as simulated equipment), and teaching materials, require financial support. Although multiple studies have demonstrated the effectiveness of simulation in the teaching of basic science and clinical knowledge, procedural skills, teamwork, and communication, as well as assessment at the undergraduate and graduate medical education levels [36], advanced simulators were not popularized in hospitals in China due to insufficient funding and technical support [37]. The pedagogical atmosphere at the ward is another factor influencing student nurses’ motivation to choose nursing as a career [36]. The positive learning atmosphere allows students to have more positive relationships with other team members, to feel genuinely involved in ward activities, and to be more motivated to explore new skills in clinical practice [37]. Therefore, we suggest increasing economic investment, establishing a teaching atmosphere, and improving departmental conditions to enhance the quality of teaching.

Interestingly, we found that among the first-level indicators, the weight of the “outcome indicator” was the smallest, and all the weights of the third-level indicators were relatively small. Medicine is an applied discipline with solid practicality. Nursing competence directly affects nursing quality and patient safety. Only with good nursing competence can they better serve patients. As with previous studies, student learning outcomes were deemed to be an important indicator of high-quality teaching [30, 38]. Therefore, “Nursing competence of the teaching object” and “Examination scores of teaching subjects” ranked high in the second-level indicators of “Outcome indicator”. However, we should also realize that students’ performance is not only related to the teacher but also to the students themselves. Perhaps due to this reason, the weights of these two indicators are slightly lower among all the second-level indicators. The weight of “Annual teaching workload” takes third place, indicating that when evaluating the clinical teaching quality, nursing managers fully recognized the impact of the department’s annual teaching workload, such as the number and duration of different teaching objects received by the department, teaching workload, examination workload, etc., and fully recognize its labor value. In addition, attention should be paid to teaching evaluation results, such as the satisfaction evaluation of teaching objects towards departments/teachers and the evaluation of teaching management personnel towards teachers, to evaluate clinical teaching work comprehensively and improve teaching quality continuously.

## Limitations

Our study has some limitations. First, due to funding and the time limit of the study, we only selected 18 experts from four tertiary hospitals and two nursing colleges in Beijing, China, to conduct the Delphi survey. Second, we used the Delphi and Analytic Hierarchy Process to construct the evaluation index system for clinical nursing practice teaching quality. These methods heavily rely on the subjective judgment of experts, which may lead to unstable and one-sided results, and lack face-to-face communication, which may result in the loss of other perspectives. Therefore, the study’s results may be biased. Third, the indicator system established in this study has not been tested for reliability, validity, and empirical application.

## Conclusions

This study established an evaluation index system of clinical nursing practice teaching quality, which included 3 first-level indicators, 10 second-level indicators, and 29 third-level indicators. The managers’ focus has shifted from outcome-oriented to process-oriented approaches. Among the second-level indicators, the experts regarded “Teaching staff”, “Implementation of teaching rules and regulations”, and “Teaching plans” more important than other indicators. Given their importance in teaching quality evaluation, more focus on teaching team construction, teaching regulations implementation, and teaching design is needed to improve clinical teaching quality. In future studies, we will continue to obtain feedback from a broader sample of experts from different regions to improve the evaluation metrics established in our study. Besides, we will design a rating scale by converting the weight value of each three-level index into a score on a 100-point scale to test the applicability and effectiveness of the evaluation index system in different contexts, and the evaluation scores could provide clues for guiding the management of clinical nursing practice teaching quality at different levels. We expect that the index system will contribute to evaluating comprehensively and improving the quality of clinical nursing practice teaching.

## Data Availability

The datasets used and/or analysed during the current study available from the corresponding author on reasonable request.
